# Respiratory variations of inferior vena cava diameter to predict fluid responsiveness in spontaneously breathing patients with acute circulatory failure: need for a cautious use

**DOI:** 10.1186/cc11672

**Published:** 2012-10-08

**Authors:** Laurent Muller, Xavier Bobbia, Mehdi Toumi, Guillaume Louart, Nicolas Molinari, Benoit Ragonnet, Hervé Quintard, Marc Leone, Lana Zoric, Jean Yves Lefrant, the AzuRea group

**Affiliations:** 1 Department of Anesthesiology, Emergency and Critical Care Medicine, Intensive Care unit, Nimes University Hospital, place du Pr Debré 30029, Nîmes, France; 2 Department of Biostatistics, UMR 729 MISTEA, Montpellier University Hospital, avenue Gaston Giraud, 34093 Montpellier, France; 3 Department of Anesthesiology and Critical Care Medicine, Nord Hospital, Assistance Publique - Hôpitaux de Marseille, Aix-Marseille University, 13015, Marseille, France; 4 Intensive Care Unit, Saint Roch Hospital, Nice-Antipolis University Hospital, 5 rue Pierre Devoluy 06000, Nice, France

## Abstract

**Introduction:**

To investigate whether respiratory variation of inferior vena cava diameter (cIVC) predict fluid responsiveness in spontaneously breathing patients with acute circulatory failure (ACF).

**Methods:**

Forty patients with ACF and spontaneous breathing were included. Response to fluid challenge was defined as a 15% increase of subaortic velocity time index (VTI) measured by transthoracic echocardiography. Inferior vena cava diameters were recorded by a subcostal view using M Mode. The cIVC was calculated as follows: (Dmax - Dmin/Dmax) × 100 and then receiver operating characteristic (ROC) curves were generated for cIVC, baseline VTI, E wave velocity, E/A and E/Ea ratios.

**Results:**

Among 40 included patients, 20 (50%) were responders (R). The causes of ACF were sepsis (*n *= 24), haemorrhage (*n *= 11), and dehydration (*n *= 5). The area under the ROC curve for cIVC was 0.77 (95% CI: 0.60-0.88). The best cutoff value was 40% (Se = 70%, Sp = 80%). The AUC of the ROC curves for baseline E wave velocity, VTI, E/A ratio, E/Ea ratio were 0.83 (95% CI: 0.68-0.93), 0.78 (95% CI: 0.61-0.88), 0.76 (95% CI: 0.59-0.89), 0.58 (95% CI: 0.41-0.75), respectively. The differences between AUC the ROC curves for cIVC and baseline E wave velocity, baseline VTI, baseline E/A ratio, and baseline E/Ea ratio were not statistically different (*p *= 0.46, *p *= 0.99, *p *= 1.00, *p *= 0.26, respectively).

**Conclusion:**

In spontaneously breathing patients with ACF, high cIVC values (>40%) are usually associated with fluid responsiveness while low values (< 40%) do not exclude fluid responsiveness.

## Introduction

Assessment of fluid responsiveness remains a daily therapeutic challenge in spontaneously breathing critically ill patients with acute circulatory failure (ACF) [1]. In mechanically ventilated patients, one of the best ways to assess fluid responsiveness is to quantify respiratory variation of arterial pulse pressure or aortic velocities recorded by esophageal Doppler or echocardiography (dynamic indices) [2-5]. However, dynamic indices are not valid in spontaneously breathing patients [6,7]. Static preload indices like central venous pressure (CVP) do not represent a reasonable alternative for two main reasons. First, central filling pressures are not systematically available in the initial phase of shock because a central venous catheter is not always available. Second, it has been clearly shown that static indices do not accurately predict fluid responsiveness, except for values < 5 mmHg [8-11]. Therefore, fluid challenge is often used to test fluid responsiveness [12]. Nevertheless, about 50% of fluid challenges are not justified [2]. This exposes patients to deleterious fluid overload. The passive leg-raising (PLR) test has been developed as a non-invasive technique to perform fluid challenge. By mobilizing the venous blood content of the leg, PLR mimics a 300 ml fluid infusion that accurately predicts fluid responsiveness [13,14], even in spontaneously breathing patients [15]. However, in case of severe pelvic or leg trauma, the PLR test cannot be performed. We recently proposed using a 100 mL fluid challenge to test fluid responsiveness in order to avoid fluid overload, but this was validated only in mechanically ventilated patients [16].

Use of respiratory IVC diameter variation (cIVC) is very popular because it is very easy to record, and needs a short learning curve, even for non-cardiologist residents or physicians [17]. cIVC has been shown to accurately predict fluid responsiveness in mechanically ventilated critically ill patients [18-20]. As with any dynamic parameter, there could be objection to using cIVC in patients with spontaneous ventilation. Nevertheless, in spontaneously breathing patients, cIVC is widely used because it correlates to CVP even if CVP is, however, poorly predictive of fluid responsiveness [21,22]. cIVC is correlated to fluid removal after chronic dialysis in nephrology outpatients [17,23], or during continuous hemofiltration in non-ventilated ICU patients with acute severe heart failure [24]. The monitoring of blood volume is not the same as evaluating fluid responsiveness, but there is a risk of confusing the two concepts. In clinical practice, physicians can then use cIVC to predict fluid responsiveness in spontaneously breathing patients because it correlates with blood volume. Therefore, it can be questioned if cIVC diameter can effectively predict fluid responsiveness in spontaneously breathing patients and if there are limitations to this technique.

Therefore, the present study was aimed at assessing the usefulness of cIVC recorded by transthoracic echocardiography (TTE) to predict fluid responsiveness in spontaneously breathing critically ill patients with acute circulatory failure.

## Materials and methods

### Patients

This observational study was approved by our local institutional review board (Nîmes University hospital review board, reference number 110702). It was stated that informed consent was not necessary; nevertheless, the patients or their relatives were orally informed, in accordance with French legislation.

The study was conducted in a 16-bed ICU of a university hospital within a 24-month period (April 2009 to April 2011). Forty patients with ACF were prospectively included within the study period. ACF was defined as mean arterial pressure (MAP) < 65 mmHg, urine output < 0.5 mL/Kg/h, tachycardia, mottled skin and/or biological signs of hypoperfusion (arterial blood lactate > 2 mmol/L). We excluded patients in whom fluid challenge would be deleterious: those with clinical evidence of pulmonary edema, echocardiographic evidence of right ventricular (RV) failure (right telediastolic ventricle area to left telediastolic ventricle area ratio > 1) [25] or echocardiographic evidence of elevated left atrial pressure (mitral inflow early (E) wave to atrial (A) wave ratio > 2) [26-28]. The decision was based on the opinion of the senior physician in charge of the ICU.

### Measurements

For each patient, the following data were recorded: diagnosis, age (years), weight (Kg), height (cm), Acute Physiology and Chronic Health Evaluation (APACHE)-II score at admission, MAP (mmHg), heart rate (HR, bpm) and CVP (mmHg) when available.

Echocardiographic measurements were performed by four trained (level 3 [29]) operators (LM, XB, MT, GL), for whom the intra- and interobserver variability for the velocity time index (VTI) = 4 and 5%, respectively [16]), using a Vivid S6 machine, General Electrics (GE Healthcare, Chalfont St Giles, UK).

IVC was observed by a subcostal long axis view. In order to differentiate the aorta and IVC, the junction between the IVC and the right atrium was systematically assessed. A pulse wave Doppler of the IVC was also recorded in order to verify the presence of a typical venous flow spectrum. A time-motion record of the IVC diameter was generated by M-mode imaging at 2 to 3 cm from the right atrium [18,30]. Maximum and minimum IVC diameters (Dmax and Dmin, respectively) were measured over a single ventilatory cycle. The IVC collapsibility index (cIVC) was used as the primary endpoint [31]. This method was previously validated in spontaneously breathing patients undergoing renal replacement therapy [24]. The cIVC was defined as follows:

cIVC = (Dmax-Dmin)/Dmax.

cIVC was expressed as a percentage. In addition, to be sure that the formula:

(Dmax - Dmin/((Dmax + Dmin)/2 = cIVC_2_)

could not be more informative, we also built its respective receiver operator characteristic (ROC) curves.

The VTI was recorded by pulse waved Doppler on a five-chamber apical view [32]. For each step of the study, the VTI (cm) was measured in triplicate. The obtained values were averaged for its determination.

In parallel, the left ventricular filling pressures were assessed using the mitral inflow coupled to tissue Doppler imaging. The transmitral diastolic inflow, or E/A velocity ratio (velocity of the E wave/velocity of the A wave in cm/s) was recorded by pulse Doppler in the apical four-chamber view at the distal extremity of the mitral leaflets [26,33]. In the same view, protodiastolic tissue Doppler velocity was recorded at the lateral annular mitral annulus (Ea wave, cm/s) [33]. The ratio between E and Ea wave velocities (E/Ea ratio) was calculated as an index of left ventriclular filling pressure [33,34].

Right ventriclular dilatation was defined as a right to left telediastolic ventriclular area ratio > 1 (RV/LV area ratio) [25]. Left ventriclular systolic function was visually quantified as previously described [35]. Lastly, the shortening diameter fraction was determined in the M mode and parasternal long axis view.

### Protocol

After ruling out the exclusion criteria, a first echocardiography was performed in all spontaneously breathing patients with ACF. At this time (T_0_), HR, MAP, E, A, and Ea velocities, E/A ratio, E/Ea ratio, and subaortic VTI were recorded. Then a fluid challenge was performed with 500 mL of a 6% 130/0.4 hydroxyethylstarch solution (Voluven^® ^, Fresenius-Kabi, Louviers, France) infused over 15 minutes. After this fluid challenge (at T_15_), HR, MAP, E, A, and Ea velocities, E/A ratio, E/Ea ratio, and subaortic VTI were recorded. Fluid responsiveness was defined as an increase in the subaortic VTI ≥ 15% after the fluid challenge. This served to split the patients into responders (R) and non-responders (NR) [15,16,36]. Of note, the investigators were not blinded.

### Statistical analysis

Data are expressed as medians with the 5^th ^and 95^th ^percentiles. For the comparisons between R and NR, Mann-Whitney, Chi square and Fisher exact tests were performed when appropriate. ROC curves were constructed to evaluate the ability of cIVC to predict fluid responsiveness. When the AUC was greater than 0.5, the best cutoff value was defined by the closest value to the Youden index [37]. ROC curves of E wave velocity, E/A ratio, E/Ea ratio, and CVP were compared to the ROC curve of the cIVC for each individual using the Hanley test [38]. Statistical analysis was performed using SAS v 8.1 software (SAS Institute, Cary, NY, USA). All *P*-values were two-tailed and a *P*-value < 0.05 was considered significant. We assumed that cIVC would be clinically relevant if the 95% confidence interval (CI) of its area under the curve (AUC) was > 0.75, corresponding to an AUC of a good clinical tool as reported by Ray *et al. *[39]. For this purpose, 39 patients had to be included. A bootstrap analysis was used to calculate precise confidence intervals. Bootstrapping is a method for assigning measures of accuracy to sample estimates and allows estimation of the sampling distribution [40].

## Results

Among 40 spontaneously breathing patients with ACF included in this analysis, 20 (50%) responded to the fluid challenge. Regarding demographics and disease severity, no difference was observed between R and NR (Table 1). The causes of the ACF are detailed in Table 2.

**Table 1:** Characteristics of the general population and comparison between responders and non-responders at baseline (before fluid challenge)

	All patients (*n *= 40)	Responders (*n *= 20)	Non-responders (*n *= 20)	*P*-value
Age, years	63 (56, 70)	61 (49, 70)	66 (53, 75)	0.58
Weight, Kg	72 (65, 77	67 (63, 76)	76 (63, 88)	0.14
Height, cm	169 (164, 173)	170 (162, 176)	168 (160, 173)	0.38
APACHE II score	17 (14, 23)	18 (14, 29)	14 (11, 21)	0.30
Heart rate, bpm	101 (91, 116)	101 (91, 125)	103 (79, 121)	0.78
Mean arterial pressure, mmHg	71 (66, 77)	70 (61, 88)	72 (65, 87)	0.56
LVEF, %	55 (50, 60)	55 (50, 60)	55 (47, 60)	0.41
Velocity time index, cm	16 (14, 18)	14 (12, 16)	17 (15, 21)	< 0.01
E velocity, cm/s	75 (70, 80)	65 (53, 76)	82 (75, 93)	< 0.01
E/A velocity ratio	0,9 (0.8, 1.1)	0,8 (0,6, 1,1)	1,0 (0,8, 1,4)	< 0.01
Ea velocity, cm/s	12 (10, 13)	12 (9, 14)	11 (9, 15)	0.79
E/Ea velocity ratio	6 (5, 8)	5 (5, 10)	7 (5, 8)	0.40
cIVC, %	34 (16, 64)	64 (28, 100)	19 (5, 35)	< 0.01

Data are expressed in medians with 5^th ^and 95^th ^percentiles. APACHE: Acute Physiology and Chronic Health Evaluation; cIVC, collapsibility index of the inferior vena cava.

**Table 2:** Causes of acute circulatory failure

Pathology	Number of patients (%)
**Sepsis**	24 (60)
Intra-abdominal infection	10
Pulmonary infection	9
Pyelonephritis	5
**Bleeding**	11 (28)
Postoperative	7
Trauma	4
**Dehydration**	5 (13)

The total percentage is different from 100% because specific percentages were rounded.

**Figure 1:**
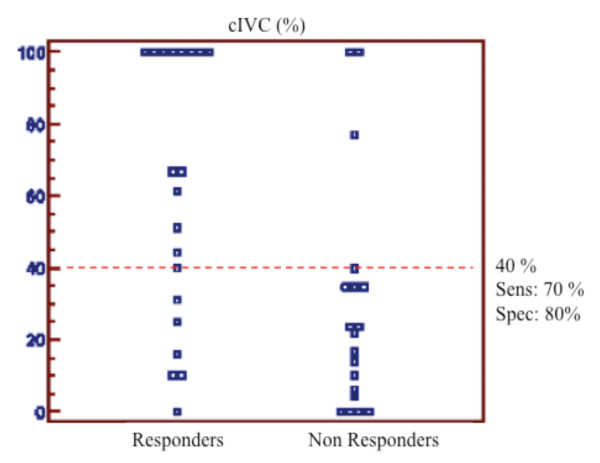
**Individual values of inferior vena cava collapsibility (cIVC) (%) after infusion of 500 mL of HES**. The best cutoff value is 40%.

**Figure 2:**
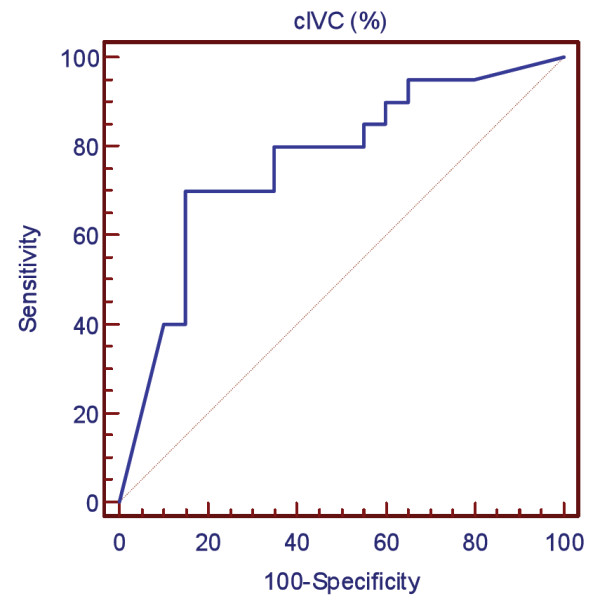
**Receiver operator characteristic (ROC) curve for inferior vena cava collapsibility (cIVC) (%) after infusion of 500 mL of HES**. Area under the ROC curve was 0.77 (95% CI 0.60, 0.88).

Individual values of cIVC according to the fluid responsiveness are shown in Figure 1. The AUC of the ROC curve for cIVC was 0.77 (95% CI 0.60, 0.88, *P *= 0.08 compared to 0.5) (Figure 2). The best cutoff value was 40%. For cIVC, the positive predictive value, negative predictive value, positive likelihood ratio, and negative likelihood ratio was 72%, 83%, 4.67, and 0.35, respectively. For cIVC, accuracy was 0.75 and Youden's index was 0.5. The AUC for baseline E wave velocity was 0.83 (95% CI 0.68 to 0.93, *P *= 0.07 compared to 0.5). For E wave velocity, the best cutoff value was 0.7 (sensitivity 67%, specificity 90%), and the positive predictive value, negative predictive value, positive likelihood ratio, and negative likelihood ratio was 84%, 83%, 6.67, and 0.37, respectively. For E wave velocity, Youden's index was 0.64 and accuracy 0.88.

The AUC for the VTI, E/A ratio, and E/Ea ratio was 0.78 (95% CI 0.61, 0.88), 0.76 (95% CI 0.59, 0.89), and 0.58 (95% CI 0.41, 0.75) respectively. There was no difference between the AUC of the ROC curve for cIVC and E wave velocity, VTI, E/A ratio or E/Ea ratio (*P *= 0.46, 0.99, 1.00, and 0.26, respectively).

Because the data set can be considered as low, and to validate our CIs, we completed the statistical analysis with a bootstrap technique. This technique accurately predicted the rate and statistical significance of the AUC difference on 1000 bootstrapped samples from the original study population. This shows a CI for cIVC = 0.59 to 0.90, median 0.83, and for E wave velocity, CI = 0.68 to 0.95, median 0.77. Bootstrap analysis tends to confirm our basic results.

When using the formula (Dmax - Dmin/((Dmax + Dmin)/2) (cIVC_2_), the AUC of ROC curve for cIVC_2 _was 0.77 (95% CI 0.60, 0.88). The best cutoff value was 25%. The AUC of the ROC curve for baseline E wave velocity, VTI, E/A ratio, and E/Ea ratio were 0.83 (95% CI 0.68, 0.93), 0.78 (95% CI 0.61, 0.88), 0.76 (95% CI 0.59, 0.89), and 0.58 (95% CI 0.41, 0.75), respectively. There were no statistical differences between the AUC for cIVC_2 _and E wave velocity, VTI, E/A ratio, and E/Ea ratio (*P *= 0.46, 0.99, 1.00, and 0.26, respectively).

## Discussion

Because the AUC of the ROC curve for cIVC was 0.77 (95% CI 0.60, 0.88), the present study shows that cIVC cannot reliably (inferior limit of CI < 0.75) predict fluid responsiveness in spontaneously breathing patients with ACF. More precisely, a cIVC value below 40% cannot exclude fluid responsiveness while patients with cIVC above 40% are more likely to respond to fluid challenge. The 40% cutoff value is in agreement with recent studies [22].

The first explanation for these imperfect results is that, as previously suggested, cIVC is a dynamic preload index. In contrast with findings reported in mechanically ventilated septic patients, dynamic parameters have been shown to be ineffective to predict fluid responsiveness in spontaneous breathing patients [6,7]. Spontaneous ventilation implies a very wide range of breathing patterns. In patients with spontaneous ventilation, respiratory variations are highly variable from one cycle to another in a given patient and between different patients. Then, influence of breathing pattern on cIVC is also variable. The present results indirectly confirm that spontaneous breathing is a natural limit for the use of a dynamic parameter.

Because previous studies have reported a good correlation between cIVC and blood volume removal during hemodialysis [17,24] or during blood donation [41], the inability of cIVC to predict fluid responsiveness may be surprising in spontaneously breathing patients with ACF. However, monitoring blood volume during blood removal is not the same as predicting fluid responsiveness. It has been shown that there is a good correlation between high cIVC value and low CVP value [21,22,42]. A low CVP value (< 7 mmHg) could be considered a good indicator of fluid responsiveness [11], corresponding to high values of cIVC (specificity = 80%). In contrast, lower values of cIVC values are poorly predictive, corresponding to higher values of CVP [8,9].

The conditions of measurement of cIVC could be discussed. In the present study, the IVC diameter was measured by M mode at 2 or 3 cm from the right atrium, as described in previous studies [17,18,22]. However, Wallace *et al. *[43] recently showed that in spontaneously breathing healthy volunteers, variations of IVC diameter were significantly lower when recorded closed to the right atria (cIVC = 20%) than when recorded 2 cm caudal to the hepatic vein inlet (cIVC = 30%, *P *= 0.03) or at the level of the left renal vein (cIVC = 30%, *P *= 0.002) [43]. This finding would explain our high rate of false negative results. This hypothesis needs to be tested in further studies. A second methodological concern is that cIVC may be influenced by the magnitude of respiratory movements, especially in the case of dyspnea, a typical feature in patients with circulatory failure and/or shock. As discussed above, the wide range of breathing patterns observed in spontaneously breathing critically ill patients is probably confusing. Kimura *et al. *[44] recently showed that breathing manner significantly affects cIVC in spontaneously breathing volunteers. This could explain why three patients in the present study showed high cIVC values without response to fluid challenge, but this hypothesis cannot actually be verified.

The choice of the formula for cIVC could also be debated. As described in the method section, we used the cIVC formula (Dmax - Dmin/Dmax). One could argue that the cIVC formula used by an other group [18] (cIVC_2_) could better analyse the variability of IVC ventilatory variations. After testing the two formulas, we did not observe any difference between the two indices. Then, the type of formula is not a major determinant of IVC respiratory variation analysis.

Mitral Doppler inflow patterns allow indirect assessment of left ventricular filling pressure [26]. In particular, E wave velocity is correlated to patients with pulmonary capillary wedge pressure [26,27]. In outpatients with preserved systolic function but significant ischemic or hypertensive heart disease, low (< 60 cm/s) or high (> 90 cm/s) E wave velocities are correlated with low and high left ventricular end diastolic pressure (LVEDP), respectively [45]. Similarly, our findings show that baseline E wave velocity was also significantly lower in R at 65 cm/s (53, 76) than in NR patients at 82 cm/s (75, 93) (*P *= 0.0005). Even if it was not the primary objective of this study, this suggests that E wave velocity < 70 cm/s (best cutoff value) could help to identify responders A spontaneously breathing patients.

### Study limitations

The present study has some limitations. First, the physicians were not blinded. Second, the patients were not consecutive. Indeed, to be included, the study required the presence of an eligible patient and the presence of a physician certified in cardiac echography. As most patients admitted to our ICU were mechanically ventilated, 2 years were needed to complete the present study. Third, the PLR test could be used in order to avoid unnecessary fluid infusion. Performing a PLR test with echocardiography to assess fluid responsiveness is validated in spontaneous breathing patients [15]. In the present study, a PLR test was not used because it is not a routine test in our ICU. Finally, the heterogeneous population of patients may have affected our findings. We cannot exclude that cIVC could be more or less accurate in a specific population of patients such as those with trauma or sepsis.

In summary, cIVC moderately predicted fluid responsiveness in spontaneously breathing patients with ACF. In patients with a low cIVC value (< 40%), fluid responsiveness cannot be excluded, while patients with cIVC above 40% are more likely to respond to fluid challenge. Then, despite its simplicity of use, cIVC should be used with caution in spontaneously breathing patients with ACF. Additionally, our results also suggest that low values of E wave velocity (< 0.7 m/s) could be used to identify responders to fluid challenge.

## Conclusions

In spontaneously breathing patients with ACF, despite its apparent simplicity, cIVC should be interpreted with caution. A high cIVC value (> 40%) is usually associated with fluid responsiveness while low values (< 40%) do not exclude fluid responsiveness.

## Key messages

• As demonstrated in controlled mechanical ventilation, large respiratory variations (> 40%) of inferior vena cava diameter are usually associated with a positive response to fluid challenge in spontaneous breathing patients.

• In contrast to what was demonstrated in controlled mechanical ventilation, low variations (< 40%) of IVC diameter cannot rule out a need for fluid therapy in spontaneously breathing patients with acute circulatory failure.

• In such situations, a low value of E wave velocity (< 0.7 m/S) is usually associated with positive response to fluid challenge.

## List of Abbreviations

ACF: acute circulatory failure; APACHE: Acute Physiology and Chronic Health Evaluation; AUC: area under curve; CI: confidence interval; cIVC: respiratory variation of inferior vena cava diameter, collapsibility of inferior vena cava diameter; CVP: central venous pressure; HR: heart rate; ICU: intensive care unit; LV: left ventricle; LVEDP: left ventricle end diastolic pressure; MAP: mean arterial pressure; NR: non-responders; PLR: passive leg raising; R: responders; ROC: receiver operating characteristic; RV: right ventricle; TTE: transthoracic echocardiography; VTI: velocity time index.

## Competing interests

The authors declare that they have no competing interests.

## Authors' contributions

LM conceived the study, wrote the manuscript and performed some echocardiography exams. JYL was the director of this research project and participated in the writing of this manuscript. XB, MT and GL performed some echocardiography exams. NM was responsible for the statistical analysis. BR, HQ and ML significantly helped to draft the manuscript. LZ checked the English language. All authors read and approved the final manuscript.
